# Correspondence on “Artificial intelligence as a surgical advisor before a DIEP breast reconstruction. A blinded comparative study of three large language models”

**DOI:** 10.1016/j.jpra.2026.01.048

**Published:** 2026-02-04

**Authors:** Olivier Mathieu, Marion Goutard, Jean-François Honart, Nicolas Leymarie

**Affiliations:** a Department of Plastic Surgery, Gustave Roussy Cancer Campus, 114 Rue Edouard Vaillant, 94800, Villejuif, France; b Department of Plastic Surgery, Hôpital Saint-Louis, Assistance Publique - Hôpitaux de Paris, 1 Avenue Claude Vellefaux, 75010, Paris, France

*Dear Editor*,

We read with great interest the recent article by Tabrisi et al. published in *JPRAS Open*, which provides a rigorous blinded comparison of three large language models (LLMs) for preoperative counseling before DIEP breast reconstruction.^1^ The authors should be commended for highlighting both the potential and the important limitations of current generative AI systems in patient-facing communication.

A central conclusion of their study—that no single LLM is uniformly reliable across accuracy, informativeness, and readability—has critical implications for clinical translation. While comparative evaluations of model performance are essential, they also raise a broader and increasingly relevant question: how can LLM-based tools be implemented safely and ethically in real-world breast reconstruction pathways?

Recent narrative and systematic reviews have emphasized the rapidly expanding role of artificial intelligence across breast surgery, spanning imaging, surgical planning, complication prediction, and patient-reported outcomes.^2^^,^^3^ Within this spectrum, conversational AI and chatbots have emerged as attractive tools for patient education, particularly given their ability to deliver information in accessible language and on demand. However, multiple studies have consistently shown that open-access LLMs, while capable of generating coherent summaries, frequently produce superficial responses, omit clinically relevant nuances, or generate erroneous or fabricated references when used without oversight.^4^ These shortcomings are particularly concerning in the context of breast reconstruction, where patient understanding directly influences expectations, satisfaction, and shared decision-making.

Beyond issues of factual accuracy, the integration of AI into patient communication raises important relational concerns. Trust remains a cornerstone of the patient–physician relationship, especially in oncologic and reconstructive settings. In a large vignette-based study, Zondag et al. demonstrated that AI-supported decision-making may negatively affect perceived physician competence and integrity in high-risk scenarios, particularly among female patients.^5^ These findings suggest that unsupervised or poorly contextualized AI tools risk undermining, rather than supporting, therapeutic relationships if their role is not clearly framed and governed.

In this context, we would like to highlight an institutional approach currently implemented at our comprehensive cancer center. Rather than relying on general-purpose LLMs, we have developed a hospital-based AI chatbot specifically designed for breast surgery and breast reconstruction patients. Rather than relying on general-purpose LLMs, this model is based on a hospital-developed AI chatbot designed specifically for breast surgery and reconstruction patients. The system combines a generative AI engine with a medically curated and validated knowledge base, and is intentionally constrained to deliver general, non-prescriptive information. Importantly, it operates without user identification, does not collect or store personal data, and is deployed within an infrastructure compliant with European data protection regulations and health data hosting standards.

This approach directly addresses several concerns raised by Tabrisi et al. First, anchoring responses to clinician-approved content mitigates the risk of inaccurate or misleading information highlighted in prior evaluations of ChatGPT and similar systems.^4^ Second, hospital governance clarifies accountability, ensuring that responsibility for educational content remains with the clinical team. Third, strict data protection measures—including the absence of user identification, non-collection of personal data, and deployment within health data–compliant infrastructures—address ethical and regulatory concerns that often hinder AI adoption in Europe.

Notably, recent reviews on AI in breast reconstruction emphasize that patient education represents one of the most promising yet sensitive domains for generative AI, precisely because benefits in accessibility must be balanced against risks to trust and safety.^2^ Hospital-developed chatbots may represent a pragmatic middle ground between the demonstrated variability of open-access LLMs and the growing demand for scalable, patient-centered educational tools.

In conclusion, the work by Tabrisi et al. provides a valuable methodological foundation for evaluating LLM performance in breast reconstruction counseling. Building on these findings, we suggest that future research should increasingly focus on institution-led deployment models, governance frameworks, and patient-centered outcomes associated with clinically supervised AI tools. Moving from evaluation to implementation may be a necessary next step to ensure that artificial intelligence enhances, rather than compromises, patient education and trust in breast reconstruction care.

## Data availability statement

The data that support the findings of this study are available from the corresponding author upon reasonable request.

## Funding information

None.

## Declaration of competing interest

None declared.
